# Facilitators, barriers and potential solutions to the integration of depression and non-communicable diseases (NCDs) care in Malawi: a qualitative study with service providers

**DOI:** 10.1186/s13033-021-00480-0

**Published:** 2021-06-11

**Authors:** Chifundo Colleta Zimba, Christopher F. Akiba, Maureen Matewere, Annie Thom, Michael Udedi, Jones Kaponda Masiye, Kazione Kulisewa, Vivian Fei-ling Go, Mina C. Hosseinipour, Bradley Neil Gaynes, Brian Wells Pence

**Affiliations:** 1 University of North Carolina Project, Lilongwe, Malawi; 2 Gillings School of Global and Public Health, University of North Carolina At Chapel Hill, Chapel Hill, NC USA; 3 Malawi Ministry of Health, Lilongwe, Malawi; 4 University of Malawi, College of Medicine, Blantyre, Malawi

**Keywords:** Integration, Depression, NCD, Qualitative, Providers

## Abstract

**Background:**

Integration of depression services into infectious disease care is feasible, acceptable, and effective in sub-Saharan African settings. However, while the region shifts focus to include chronic diseases, additional information is required to integrate depression services into chronic disease settings. We assessed service providers’ views on the concept of integrating depression care into non-communicable diseases’ (NCD) clinics in Malawi. The aim of this analysis was to better understand barriers, facilitators, and solutions to integrating depression into NCD services.

**Methods:**

Between June and August 2018, we conducted nineteen in-depth interviews with providers. Providers were recruited from 10 public hospitals located within the central region of Malawi (i.e., 2 per clinic, with the exception of one clinic where only one provider was interviewed because of scheduling challenges). Using a semi structured interview guide, we asked participants questions related to their understanding of depression and its management at their clinic. We used thematic analysis allowing for both inductive and deductive approach. Themes that emerged related to facilitators, barriers and suggested solutions to integrate depression assessment and care into NCD clinics. We used CFIR constructs to categorize the facilitators and barriers.

**Results:**

Almost all providers knew what depression is and its associated signs and symptoms. Almost all facilities had an NCD-dedicated room and reported that integrating depression into NCD care was feasible. Facilitators of service integration included readiness to integrate services by the NCD providers, availability of antidepressants at the clinic. Barriers to service integration included limited knowledge and lack of training regarding depression care, inadequacy of both human and material resources, high workload experienced by the providers and lack of physical space for some depression services especially counseling. Suggested solutions were training of NCD staff on depression assessment and care, engaging hospital leaders to create an NCD and depression care integration policy, integrating depression information into existing documents, increasing staff, and reorganizing clinic flow.

**Conclusion:**

Findings of this study suggest a need for innovative implementation science solutions such as reorganizing clinic flow to increase the quality and duration of the patient-provider interaction, as well as ongoing trainings and supervisions to increase clinical knowledge.

*Trial registration* This study reports finding of part of the formative phase of “The Sub-Saharan Africa Regional Partnership (SHARP) for Mental Health Capacity Building—A Clinic-Randomized Trial of Strategies to Integrate Depression Care in Malawi” registered as NCT03711786

## Background

Mental health disorders are a leading cause of death and disability worldwide. As the fifth-leading cause of disability-adjusted life years, mental illness accounts for nearly a third of years lived with disability [1]. This problem is especially high in low and middle-income countries (LMICs), which account for approximately three-quarters of this burden [2] and where depression is the most commonly presenting mental illness [1, 3]. Nevertheless, access to mental health treatment is strikingly low, with over three-quarters of those living with mental illness in LMICs not receiving any treatment, and an even smaller proportion receiving adequate treatment [4]. The main factors contributing to lack of access to mental health services in LMICs include a lack of scale-up of mental health services, inadequate mental health providers (i.e., less than two overall mental health providers and less than one psychiatrist per 100,000 population) in low income countries, and the absence of any overall policy agenda to address mental health issues all contribute to difficulties in scaling up of mental health services [5]. In Malawi, scale up is further challenged by the way mental health treatment is managed in relation to levels of the country’s healthcare delivery system [6]. In some parts of the country, mental health care has been integrated into primary and secondary care [7], however most patients with mental health problems are only able to access care at just three central hospitals where services are focused on treating the most severe mental health disorders. Reserving mental health resources for the only most severely affected creates a gap in service across lower levels of the healthcare system, potentially leaving many individuals with less severe cases of depression undiagnosed and untreated.

The mental health treatment landscape in Malawi remains similar to that of many LMICs, and calls to integrate mental health services into NCD, maternal and child healthcare, and HIV care have increased in recent years to address respective treatment gaps [8–10]. Such calls for mental health treatment integration often rely on “task-shifting,” an evidence-based implementation strategy where primary clinicians, nurses, and community health workers are trained to screen for and treat patients for mental health disorders in addition to the provision of their routine care [11]. Recent systematic reviews of task-shifting strategies found that they are not an outright solution to workforce deficiencies, and that their acceptability and feasibility are challenged by nuances across health systems [12]. Most commonly, barriers include self-perceived clinician competence, occupational distress, and a lack of compensation for the additional provision of integrated services [13]. The present manuscript further bolsters this literature by aiming to describe the barriers and facilitators to a depression-focused task-shifting implementation strategy in Malawian NCD clinics.

The need for successfully depression/NCD integrated services is particularly acute regarding NCD treatment. There is evidence that depression results in worse NCD outcomes, and patients with NCDs are more likely to be diagnosed with depression [8, 14]. While integrating depression services into infectious disease care has proven feasible, acceptable, and effective in sub-Saharan African settings [15, 16], integration of depression services in other clinical settings are difficult, even in high income countries [17]. Accordingly, the goal of this paper is to contribute to the literature on implementation science by describing the facilitators, barriers and potential solutions to the integration of depression and NCD care in Malawi, specifically from the perspective of NCD providers. For the purposes of this paper, “depression care” refers to the use of a clinical tool to screen for depression (Patient Health Questionnaire-9), anti-depressant medication prescribed in accordance with an evidence-based algorithm, and an evidence-based psychosocial counseling intervention (the Friendship Bench) [18–20].

This study generates an additional reference point from the Malawian context into the growing literature base of barriers and facilitators to task-shifted mental health services in LMICs. The results of such studies extend beyond themselves or their immediate subsequent trials. This study, and others like it, push the fields of mental health and implementation science forward by crystalizing common challenges that impede mental health treatment integration across contexts. Higher quality implementation strategies that can address or be tailored to nuanced barriers in LMIC healthcare systems may only come to light through the maturation of the literature base to which this paper aims to contribute.

## Methods

### Design and setting

This qualitative study was part of a formative phase of a subsequent implementation science trial testing the efficacy of two implementation strategies at integrating depression and NCD care in Malawi. This formative phase specifically explored the possibilities of integrating depression care into NCD clinics that primarily serve patients with either hypertension or diabetes. We conducted this qualitative study in ten hospitals that were located within the Central Region of Malawi. Nine of the 10 hospitals were designated as district hospitals, and one was a community hospital. The Malawian health care delivery system is divided into three levels of care: (1) primary level that is provided in health centers and community hospitals, (2) secondary level that is provided in districts hospitals and Christin hospitals with capacity equivalent to a district hospital, and (3) tertiary level that is provided in central hospitals [6]. Primary care comprises of outpatient and maternity services; secondary level hospitals do provide care to patients referred from health centres and community hospitals and also serve their surrounding populations with both outpatient, maternity and services that require hospitalization. Ideally, tertiary level of care is supposed to be providing specialized health services while receiving referral s from district hospitals but in reality, they also do provide outpatient services. NCD clinics are relatively new in Malawi and have been mostly established in district and community hospitals that serve a high proportion of patients. These hospitals were all identified by the Malawi Ministry of Health (MoH) headquarters as having established NCD clinics.

### Data collection, management and analysis

We used a qualitative descriptive approach in the design, data collection and analysis of this formative study [21, 22]. The goal of using this qualitative descriptive approach was to come up with descriptions of barriers and facilitators, as well as their meaning [21], from the perspective of NCD providers regarding the effective integration of depression care into NCD care in Malawian hospitals. Between June and August 2018, we conducted 19 in-depth interviews with NCD providers at 10 Ministry of Health (MoH, government run public) hospitals in the central region of Malawi (i.e., two providers per hospital except at one hospital where we interviewed one due to scheduling problems). The interviewed providers were defined as either Clinical Directors or their designees, NCD Coordinators or their designees. The Clinical Director is a title that is given to either physicians, physician assistants/clinical officers, or nurses to lead a hospital. NCD Coordinator is a title given to either physicians, physician assistants/clinical officers, or nurses who are directly working with NCD patients and are responsible for coordination of NCD care at the designated NCD clinics. The clinical team is led by the District Medical Officer (DMO) in most district hospitals. The DMO is a physician by training. NCD clinics are staffed by general physicians, physician assistants/clinical officers and nurses who rotate throughout all units at the hospital over time. In Malawi, health care workers such as physicians, physician assistants/clinical officers and nurses are all trained as general providers in their field but can choose to specialize in various fields such as psychiatry and become mental health specialists. There is no specialization currently for someone to become an NCD practitioner in Malawi, therefore, every nurse or clinician who works at NCD clinic is referred to as an NCD provider.

Interviews were framed by a semi-structured interview guide that was developed by members of the study team. Given the background of health worker training in Malawi, which includes some curricula regarding depression diagnosis and treatment, questions on the interview guide focused on the experiences of the NCD providers on depression management, and their perspectives on the integration of depression care into their existing NCD clinics.

While the guide did not formally ask about facilitators to integration of depression treatment into NCD care, our interview guide described the process of depression care and asked about providers’ perspectives on the process. These questions and their subsequent probes naturally led to the elicitation of barriers and facilitators to the integration of depression care into their NCD clinics. Through probing, discussions during interviews regarding these topics led to identification of solutions to the identified barriers.

The interview guides were translated into Chichewa by two Malawians fluent in both English and Chichewa. Chichewa is a national language of Malawi and is a local language of all respondents in this study, while English is an official language that is mostly used in schools, offices and in all official communications. While most interviews were carried out in English, the interview guide was translated to accommodate study participants who may have wished to be interviewed in Chichewa.

Senior qualitative study staff trained two bilingual Malawian research assistants to use the interview guide and to conduct interviews. Training specifically focused on building depression and NCD knowledge, asking open-ended questions, probing, and body language. Demographic data were collected using a separate form.

All interviews were carried out at the clinics but in private rooms to ensure confidentially. All interviews were audio-recorded; and supplemented with field notes taken during or just after the interviews. To promote privacy and confidentiality, audio recordings were then transferred into an encrypted computer with passwords known by the transcriber only; then the audio recordings from the recorder were deleted. Audio recordings were transcribed and translated into English using a one-step approach (i.e., Chichewa sentences within the interview were transcribed directly into English) while field notes were expanded and used to write summaries that were fitted within the transcripts.

Thematic analysis was facilitated by Dedoose Version 8.0.42. Thematic analysis is a qualitative method focused on the development of themes to answer a specific research question; in practice this involves reading through transcripts to be familiar with the data, code development and coding, identification of possible themes, review and analysis of those themes to identify structures, and lastly checking new themes against new data [23]. To go through this analytical process, two senior qualitative study staff developed a codebook that was used to categorize and reduce the raw data. Initial codes originated from main questions on interview guides (e.g., Implementation facilitators and implementation barriers) and additional codes were added as themes emerged (e.g., emerging solutions to the identified barriers). We used both inductive and deductive approaches to identify themes that emerged as either facilitators or barriers to integrating depression into NCD clinics. Themes related to possible solutions to the identified barriers were also identified. Coded themes were further developed by coding summaries and matrices to better understand NCD providers’ attitudes towards the integration of depression care at their NCD clinics. We present themes that emerged as facilitators, barriers and potential solutions to the identified barriers to better integrate depression care into routine NCD care at the 10 public hospitals in Malawi.

### Guiding theoretical framework

This qualitative analysis was guided by the Consolidated Framework for Implementation Research (CFIR). The CFIR supplies implementation researchers with a set of five domains: *intervention characteristic* which reflects perceptions of participants describing features that are available to influence implementation; the *outer setting* which include needs of the patients, barriers and facilitators to meeting identified patients’ needs and whether these are known by the intervention implementing organizations; the *inner setting* which include features influencing interventions’ implementation specific to the implementing organization; *characteristics of individuals*, who are involved in implementation of the intervention; and *process* which includes procedures or strategies that an organization go through to implement an intervention [24]. The framework features 39 sub-constructs within its five domains to encompass the breadth of multi-level forces that might act on program implementation. Thus, the CFIR is useful during the formative qualitative evaluation phase of implementation science studies due to its ability to pre-specify and categorize barriers and facilitators to intervention implementation, thereby boosting the applicability and interpretability of the qualitative findings [25, 26]. In this analysis, we categorize our results into CFIR domains and constructs to systematically outline barriers and facilitators so that they can be more easily linked to subsequent implementation strategies during the trial phase of the study. This effort serves well for our immediate goals of informing the parent trial's implementation strategies in Malawi and in similar instances of depression integration efforts elsewhere.

### Ethical considerations

Two Institutional Review Boards (IRBs) approved this study: the University of North Carolina at Chapel Hill Institutional Review Board (Chapel Hill, NC, USA) and the National Health Science Research Committee (NHSRC) of Malawi (Lilongwe, Malawi). All study hospitals through their hospital director or designee agreed to participate in this study. All study participants provided written informed consent.

To maintain privacy and confidentiality, all IDIs were done in a private room chosen by the NCD providers within the hospital. Interviewees were identified by numbers on all interviews. Audio recordings were transferred into an encrypted computer with two passwords known by the transcriber only. Thereafter, all audio recordings in the recorder were deleted.

## Results

Our findings are arranged in two parts: demographic characteristics of the study respondents, and the main findings comprised of themes that emerged from the data. We categorize our main findings into four CFIR domains: intervention characteristics (relative advantage and design quality and packaging); characteristics of individuals (knowledge and beliefs about the intervention); outer setting (patients’ needs and resources); and inner setting (readiness for implementation).

### Demographic characteristics

Of the 19 respondents of this study, three were females and 16 males. Professionally, 18 were either physicians or physician assistants (clinical officers), and one was a nurse. Of these, nine were District Medical Officers who were interviewed as designees of the hospital directors while 10 were NCD coordinators.

### Overview of key findings

Respondents described features of the hospitals that may influence implementation of an integrated depression and NCD care; the relative advantage of integrating depression care into NCD care and the design quality and packaging of tools that may be used to effectively integrate depression into NCD care in these hospitals. Our study found that all 10 hospitals were providing NCD care, and all but two had a dedicated room for NCD services. The respondents described depression assessment tool as easy to use and if available to the hospitals, may help them to effectively assess their patients more holistically.

Knowledge and beliefs about integrating depression into NCD care were also discussed. Almost all respondents were able to define some characteristics of depression symptoms consistent with DSM-5 diagnostic criteria [27] as illustrated by the following quote:

…if we say that this one is depressed, mostly, they do not say anything, they just remain quiet…there is quietness, failure to sleep as I already said and even suicidal ideas can come in. You might find that someone is isolating himself but after you inquire closely, you might find that the person is having suicidal ideas….(NCD Provider).

All NCD Providers who participated in this study also reported that they had encountered patients with depression symptoms at the NCD clinic. Almost all respondents reported that integrating depression care into NCD care was feasible. Limited knowledge and lack of training regarding depression care was identified as a barrier to integration of depression care into NCD care in these hospitals.

Patient needs and resources were discussed and fell into barriers related to the outer setting domain of the CFIR, mostly relating to the availability of antidepressants at the hospitals, inadequacy of both human and material resources, high workload experienced by the NCD providers, and a lack of physical space for depression services (especially counseling services).

Providers also discussed staff shortages given that providers are assigned other hospital duties in addition to their work within the NCD clinic. Staff shortages relate to the implementation climate CFIR domain. While staff rotations were thought to lead to a shortage of NCD providers, providers’ willingness to help patients with depression, emerged as a factor to facilitate depression integration into NCD clinics.

Out of the identified barriers, respondents suggested the following as possible solutions: training of NCD staff on depression care, engaging all staff who are involved in NCD care in depression care training, engaging hospital directors and managers to create an NCD and depression care integration policy, integrating depression information into existing NCD documentation, increasing staff, and reorganizing clinic flow to reduce patient wait times. The following section describes in detail, the facilitators, barriers, and solutions to integrate depression into NCD care in Malawi.

### Facilitators to the integration of depression and NCD care

#### Characteristics of the individuals

This study found that most hospitals have experienced physicians and physician assistants who understand the importance of treating depression. In addition, most hospitals in Malawi have providers with specialized training in mental health, who are already assisting patients with comorbid of mental health disorders and NCD conditions.

In the following quote, one NCD provider emphasized how integrating depression into NCD care would help identify patients who are struggling with NCD management and may be suffering silently:

“As [x hospital], I think if the integration of NCD and depression is implemented, I see benefits to our patients because most of them have been complaining about the chronicity of their conditions… because if a hypertensive person has been taking drugs for a long time, we are not expecting such an individual to stop taking treatment [of hypertension]. Some of them also complain even though we explain that with their condition they cannot stop taking medication. There are also others who don’t openly complain but they complain silently. So, if we can integrate assessment of depression I think we will also uncover those that are not complaining, those that are silent” (NCD provider).

Another provider talked about the importance of integrating depression care into NCD care that may help to reduce depression symptoms in NCD patients:

“...I think the program [of depression care], if it is to be implemented and incorporated into the NCD clinic, it would be a good program and it would also improve the treatment outcomes of our patients and also help to reduce problems of depression regarding chronic conditions” (NCD, Provider).

#### Readiness for implementation

Willingness by the NCD providers to integrate depression care into their existing NCD clinics is an indication that there is some level of readiness for integration on the part of the NCD providers at the study hospitals. Almost all respondents (18/19) reported that integrating depression care into NCD care is realistic, and feasible. The following quote illustrates the readiness of NCD providers to integrate depression care into their NCD clinics.

“My thoughts are that there is need for these things [NCD and depression care] to be integrated so that these services should run together because it is the same people that have come that have depression and the same ones that have NCDs” (NCD Provider).

#### Intervention characteristics

NCD providers reported that the Patient Health Questionnaire-9 (PHQ-9) sounded feasible and easy to use. This low level of perceived difficulty relates directly to the CFIR construct of complexity. The providers reported that use of the PHQ-9 would help them to identify patients with depression who could not have been identified during their routine NCD care. NCD providers also reported that integrating depression assessment and treatment into NCD care would help them to assist their patients more holistically, relating to CFIR construct of relative advantage.

“To me I feel like it’s possible; because since it will be like we have a checklist, so the checklist also helps a lot so that you don’t miss other things. Yeah. And also the way am saying it that not all [patients] are able to express their problems once they are here, so as we are assisting them…that if we are to have the system that…we’ll be asking them those questions it will be very helpful for us that maybe we will be able to tell on how many have come with depression unlike the way we are doing it right now; maybe we do not do much of a research that we should take note on how many of them have depression. Yeah. So, it can be very helpful” (NCD Provider).

Another NCD provider supported the potential ease of use of the PHQ-9 tool stating that:

“Nine questions are not a lot. It’s just a small questionnaire that you can use almost on every patient. We can do that without any difficulties…” (NCD Provider).

#### Outer setting

All study hospitals had amitriptyline as an antidepressant in stock within their pharmacies at the time of interviews, but these antidepressants were reported to be mostly prescribed by providers with mental health specialization and not the general NCD providers. Therefore, amitriptyline could be reallocated when integrating depression care into NCD care. The following quote report how NCD clinicians work with mental health clinicians.

“because here at NCD [clinic], we deal with diabetes but when it comes to depression, we refer to Mental Health staff…I can give her/him the medications that can help to control the NCD conditions. For this one with depression…I can treat her/his NCD conditions and then refer to mental health staff so that they can provide counseling or treatment [antidepressants] for the depression.” (NCD Provider).

Another NCD clinician described how prevalent depression is and how it is treated at mental health clinic:

“At the OPD [outpatient department], and a lot of them come that side [at mental health clinic]. When we are compiling psych[iatric] data, we have several people under the category of depression. We have several people who are on anti-depressants.” (NCD Provider).

#### Inner settings: structural characteristics

NCD providers discussed how harnessing their routine division of clinical labor could facilitate the integration of depression care. The NCD providers described how they would use different team members to share the integrated depression screening and treatment activities while continuing to manage patients’ NCD conditions in-step with their current duties. The following is the direct quote of a NCD provider who stated how the available team would work together to integrate depression care into NCD care:

“I think the possibility [of integrating depression care into NCD clinic] is there. Because, I am looking at our clinic, we have someone who checks the blood pressure…there are three of us. Someone will be checking the blood pressure, a clinician doing the consultations and someone else doing the dispensary of drugs…we can allocate [someone to screen for depression], of course that would most likely be done by the clinician.” (NCD Provider).

### Barriers and possible solutions to depression care integration

Barriers are issues that were reported by NCD providers to potentially hinder integration. Emerged barriers to integration of depression services into NCD care relate to the CFIR domains of characteristics of individuals (perceived knowledge and beliefs about the intervention) and the outer setting (patient needs and resources).

Most NCD providers reported limited knowledge and lack of training regarding depression care, inadequate resources including staff shortages and shortage of general NCD drug supplies (e.g., antihypertensive and diabetes drugs), high workload and perceived additional work that might lead to long patient wait times, and a lack of physical space for depression care.

Possible solutions to the identified barriers included training the NCD providers to assess and treat depression, engaging hospital directors and managers to create an integrated NCD/depression policy that may guide NCD providers on how to implement integrated care, integrating depression information into existing NCD documents, increasing staff at the NCD clinics, re-organizing clinic flow, and providing integrated services at community levels.

#### Characteristics of individuals: knowledge and beliefs about the intervention

Limited depression care knowledge and training represented a salient barrier for the NCD providers. Although the general Malawian academic curricula for medical professionals does include some training on general mental health disorders including depression, systematic assessment of such disorders using validated screening tools are not part of the curricula. Another general problem is that once health professionals graduate from their medical schools, they do not practice mental healthcare if they are not working at a specialized psychiatric health unit. In this study, almost all NCD providers who were interviewed reported inadequate knowledge on how to assess and manage depression using standardized tools. The following quote emphasizes lack of depression knowledge as stated by one NCD provider:

“Umm it’s not easy for us doctors to notice that for this person, the problem they really have is not malaria or pneumonia but maybe it is depression. It depends on the doctor’s interest for them to notice such things. Another thing is the skill or knowledge to know that this is depression, not all doctors are capable enough to know and diagnose someone as having depression. So, there is that lack in us doctors” (NCD Prover).

NCD providers also suggested training the general providers who work at NCD clinics on how to use the depression assessment tool (the PHQ-9) and orienting them to depression treatment options which would help them to integrate depression care into the NCD clinics. Since providers in Malawian hospitals frequently rotate between hospital units (e.g., NCD clinic, Maternal and Child Health clinic, etc.), training all staff who could become involved with NCD care would be preferred rather than just training some. The following quote emphasizes the importance of training the NCD providers to facilitate the integration of depression into NCD care:

“They [other NCD providers] can accept and implement it only if they are taught and oriented on what to do. Every clinician knows their work, so they can take this up without a problem…if they are told the questions which they would need to ask. We do not know exactly what you need and so we can be administering the questions anyhow but if you teach us, that would help us as clinicians to notice people with depression” (NCD Provider).

Continuous training support to district hospitals that may integrate NCD and depression services was also suggested by the NCD providers as a good approach to sustain the integrated services. As described above, because providers frequently transfer to other hospitals or rotate across units within the same the hospital. The following quote emphasizes the need for training staff to sustain an integrated depression and NCD program:

“Sustainability, let us say maybe in supporting the program, support could be holistic, would be…further training of people because people keep on changing [posts and units]. Today I would be here and tomorrow they transfer me to [another hospital or to another unit] that would mean that program would think there are people that they trained but these things happened some time back and when you come back and see the reports you find the new people are not following…” (NCD Provider).

#### Implementation climate: available resources

Inadequate resources to integrate depression care represented a common barrier described by the providers. Specifically, staff shortages, a shortage of general NCD drug supplies, and a lack of physical space were reported as the most salient resource barriers. NCD providers reported that Malawian hospitals are chronically understaffed leading to high workload across all units, including those working at the NCD clinic. NCD providers perceived that integration of depression care, in an already understaffed and overworked environment, would create additional work for the already overstretched NCD providers. The following quote emphasizes how a shortage of staff may inhibit integration of depression care into NCD care:

“It will not be easy for them [other NCD providers] to accept because it will be like you have added extra work to them. So, it will not be easy, but through the benefits that I have explained [to effectively manage NCD patients who also have depression] and by sitting down with them and explain[ing] the importance of integrating depression, they can understand. They will understand that it is very important to tackle this issue [of integrating depression into NCD care]” (NCD Provider).

NCD providers suggested that engaging hospital directors and managers to create an NCD and depression care integration policy may be one of the solutions to effectively overcoming this barrier. The suggested policy would mandate the need for clinicians already working in NCD clinics to begin providing integrated depression care. NCD providers also suggested the need to integrated depression information into existing NCD documents (e.g., the NCD Mastercard, which tracks patients’ NCD diagnoses and treatments).

“…but then I don’t know how it’s going to be like because the clinic is not a one man’s thing, for that reason I feel like…Because we do have NCD master cards so I think to make it simple or maybe for continuous mode of care, I feel like that NCD master card should be revised and it should include a field where we are to fill in the depression’s severity, yeah. That either the person has depression or doesn’t have that depression or if [the patient] has it we can then record on how severe it is on that same master card; on the same master card we should have a column where we will indicate the type of depression medication that we have given to that patient, we should be indicating that we have given them this kind of medication same as the way we do with diabetes and hypertension…” (NCD Provider).

Another suggested solution was to increase staff at the NCD clinics:

“If there was enough staff, things would work better because we already have all the necessary resources. The staff is very little. If there can be additional staff things may work out smoothly and there can be less pressure on us clinicians” (NCD provider).

From the perspective of NCD providers, shortage of anti-hypertensive or diabetic drug supplies may also hinder integration because patients may not adhere to visits to receive the NCD/depression integrated care. In Malawi when patients go to a public hospital, they expect to consult with a service provider who will prescribe drugs for them, and they will then be able to procure those drugs free of charge at a hospital pharmacy. Because prescribed anti-hypertensive or diabetic are often in short supply, NCD providers expressed concern that patients with NCD/depression conditions may not adhere to appointments. The below quotes emphasize how NCD providers feel shortage of anti-hypertensive or diabetic may affect patients’ follow up:

“…we are able to help clients but the challenge is drugs. Even if we can go to the pharmacy right now we don’t even have a single drug of diabetes…We can help the person so well, but the challenge is that we advise them to go and buy medicine themselves because we don’t have the drugs. As such they leave the clinic demotivated. As a result, next time if they are sick, they reluctantly come to the health center as they expect to be told that there are no drugs again” (NCD Provider).

Another NCD Provider added that:

“We only give them the medications that are available at that time. If we don’t have any of them that means the patient will not be assisted” (NCD provider).

To ease the problem of inadequate resources such as staff shortages and shortage of general NCD drug supplies, NCD providers suggested decentralizing NCD/depression services to the community level in order to facilitate care. Because NCD services have not been decentralized to primary care levels that are located in the communities closer to where patients live, NCD patients travel long distances to receive care at the district or community hospitals. Therefore, decentralizing NCD/depression services may help patients receive the care they need without having to incur the costs associated with travel. The quotes below emphasize what patients face by traveling long distances to receive their NCD care at either a district or community hospital:

“Yes, and it is the same with the patients that have hypertension, they come from far. Let’s say a person has come from [a village far from the hospital]…this is the only NCD clinic and every one [with NCDs] comes here, there is no other clinic. So people come from very far to get here and have come in the morning. They come with stress that they have lost money but then get the care so late” (NCD Provider).

The quote below emphasizes how providing psychosocial counseling at the community level may be sustainable, assist patients with depression, and reduce the burden patients face in traveling long distances to receive their NCD care:

“You see, in an event that drugs are not available in government hospital, counselling [if provided at community] can help as it is free, readily available in the community and can stay in the community for years. It’s sustainable” (NCD provider).

Although most NCD providers stated that their hospitals have a dedicated room or two for general NCD services, providers from two hospitals reported a lack of physical space for general NCD care: at the time of data collection for this study, a common area within the hospital was being used to provide NCD care. Use of common areas is challenging in Malawi especially during the rainy season that runs from December through March or April, forcing clinic services in-doors. Additionally, providers thought if depression services are integrated into NCD care, space may not be available for depression specific services like psychosocial counseling. Counseling would require a separate space other than the current spaces used for NCD services. Below is a quote describing how lack of physical space affects NCD work especially during rainy season:

“…We just set tables and everyone sits in their place [without minding of confidentiality of patients]. We had a tent previously but unfortunately when the rains came, the tent fell…If it is raining it is really difficult for us because you find that we go into the hospital corridors [hall ways] because the blocks we have this side are few and it is really hard. We find ourselves sitting in the corridor or maybe we will be in the dental ward although the space is small” (NCD Provider).

## Discussion

This qualitative study aimed to strengthen the literature on integration of mental health services into LMIC non-specialist clinical care by exploring barriers and facilitators to the integration of NCD and depression services in Malawi. We took a theory-informed approach to the categorization of each barrier and facilitator using CFIR domains and constructs. In our sample, we found that all 10 study hospitals were able to provide NCD care and most had a dedicated room for NCD services. NCD providers had some knowledge of the depressive symptoms as they were able to define some characteristics of depression symptoms according to DSM-5 diagnostic criteria [27]. They also reported that it is feasible to integrate depression care into NCD care, and they were ready and willing to holistically help NCD patients with co-occurring depression. However, NCD providers reported that limited knowledge and lack of training regarding screening with validated depression tools and systematic depression care, inadequacy of both human and material resources, high workload experienced by the NCD providers and lack of physical space for depression services (especially counseling services) would hinder integration of depression into NCD care at their facilities. Additional training, supervision, staffing, medication, and physical space are intuitive solutions to the barriers identified by providers. Although, in resource-constrained health systems, it is likely unfeasible and unhelpful to suggest that governing bodies provide additional funds to hospitals to increase staffing, physical space and medication. In line with the literature base, the implications of these results may be best addressed through innovative implementation science solutions [17, 28, 29] in order to integrate depression assessment and care into NCD clinics in Malawi.

The literature suggests appropriate implementation strategies to address the barriers to integration of depression treatment identified in this study. For example, limited knowledge can be addressed by training NCD providers to assess and treat depression within their clinics; thereafter, continue building their capacity through provision of ongoing technical assistance [29, 30]. Training is a planned, instructional, interactive activity that enables the learners to acquire knowledge, skills, and attitudes to improve their performance, while technical assistance is an individualized, hands-on consultation that is generally provided after training to reinforce understanding of knowledge and skills in practice settings [29–32]. If NCD providers can be trained, followed by on-going trainings, provision of technical assistance, and supervision to increase and sustain clinical knowledge of depression assessment and care, it could minimize intervention drift, leaving depression services more likely to be successfully integrated into general NCD clinics of Malawi.

Providers did not mention mental health stigma as a perceived barrier to depression care integration. This was somewhat surprising given that globally, mental illnesses are associated with stigma which frequently acts as barriers to effective treatment [33–35]. While little research exists into mental health stigma in Malawi, one study of hospital patients found very few participants willing to endorse feelings of shame if someone in their family experienced mental illness (8.1%), most endorsed the ability to maintain a friendship with someone who had been mentally ill (68.5%) [36]. Some participants in the aforementioned study had been treated for mental health disorders at a psychiatric clinic and their views may not be representative of the general Malawian population. Therefore, more studies are required to fully grasp mental health stigma in this context. A lack of mental health awareness may also play a role regarding mental health stigma in Malawi. Our research team carried out a similar qualitative study of *NCD patient* attitudes regarding depression and depression integration in tandem with this study of providers. Two patients from each of the same 10 hospitals in this study were interviewed one-on-one. Patients did not tend to describe mild or moderate depression as a medical condition, and instead thought of it more as a normal part of life [37]. Because of this, the patients described the power of their providers to shape how a patient might react to depression screening, diagnosis, and treatment [37]. Patients in another study done in Malawi described depression as looking sad or different than usual, self-isolation, ‘thinking too much’, and anger [38]. A study by Tugumisirize in 2005 focused on how psychological problem are presented in two main Malawian languages (i.e., Tumbuka widely spoken in northern Malawi and Chichewa widely spoken in central and southern Malawi) and revealed that both languages have rich vocabulary for emotional words, phrases, metaphors and idioms for expression of sadness, misery and sorrow, associated with grief and bereavement. Furthermore, it was found that the vocabulary for expression of grief and bereavement is very similar to the vocabulary for expression of depressive illness in both languages [39]. In newly diagnosed HIV positive Malawian pregnant women, depression was described as “pain in ones’ heart” [40].

Inadequate human and material resources such as staff and medication shortages, lack of mental health training, lack of physical space, and a lack of coordination within healthcare systems have been cited in literature as barriers to implementing evidence-based health interventions in LMICs [8, 41, 42]. The Malawian healthcare system is chronically understaffed in most of its units including NCD clinics and suffers from a lack of trained mental health professionals overall. “Task shifting,” or training non-specialists to provide mental health services may be harnessed to implement evidence-based interventions like depression care. Task shifting is a concept of using non-specialized staff to perform duties of specialized staff [43] and has been widely adopted in Malawi and most countries in Africa across various fields of health [43–45]. In integrating depression into NCD care, depression management interventions that do not require antidepressant medications such as counseling can be shifted to non-health workers. Studies have shown that psychosocial counseling programs can be implemented successfully using task-shifting approaches [44–46]. However, task-shifting alone may be insufficient in addressing some of the nuanced barriers described by these Malawian NCD providers. Best results may be to pair task-shifting with additional implementation strategies targeted at barriers like limited clinic staff. Providers in this study suggested reorganizing clinic flow to increase the quality and duration of the patient-provider interaction. In addition, providers suggested that hospital management need to be knowledgeable regarding the availability of antidepressants and general NCD drugs to ensure adequate supply at health facilities.

To our knowledge, this study was first to explore NCD providers’ perspectives about integrating depression care into NCD services in Malawi. However, our findings do have a number of limitations. First, our focus on front line NCD providers from public hospitals located in the central region of Malawi excluded policy makers, potentially leaving out some of the pragmatic barriers and facilitators to integrate depression assessment and care into NCD care in Malawi. Nevertheless, District Medical Officers and NCD coordinators retain a high level of influence over hospital and NCD clinic activities, and are likely able to make meaningful clinic-level impacts on their own. We therefore believe that our findings have relevance for many comparable clinical settings in LMICs.

Secondly, although NCD Providers reported that integrating depression care into their NCD clinics is feasible with suggested solutions to the identified barriers, we recognize that this decision is theoretical and needs to be tested and evaluated in order to make further recommendations. Depression care was not part of the NCD program at the time of data collection and therefore, feasibility of integrating depression assessment and care into NCD care could not be assessed.

Finally, this study used a qualitative approach that provided an in-depth understanding of contexts that may hinder or facilitate integration of depression care into NCD care in Malawi. We recognize that results of this study may only be applied to other contexts similar to the studied populations and hospitals.

## Conclusion

Our study found that NCD providers in low-resource clinical settings in Malawi already encounter patients with depressive symptoms and are willing to integrate depression care into their NCD clinics. However, successful integration of depression into general NCD clinics can be achieved in such contexts only if barriers such as a lack of knowledge and training regarding depression care by the NCD providers and insufficient human and material resources are addressed. Key considerations for the successful integration of depression into NCD care in such contexts include training NCD providers on how to assess and treat depression, engaging hospital directors and managers to create an integrated NCD/depression policy that may guide NCD providers on how to implement integrated NCD/depression services, integrating depression information into existing NCD documents, further task shifting some of the work to non-health workers such as peer NCD patients to provide psychosocial counseling, reorganizing clinic flow, and providing integrated NCD/depression services at community levels.

Our findings are relevant not only to improving the integration of NCD and depression care in Malawi, but to the improvement of similar depression integration efforts across primary and non-specialist care settings in LMICs. Calls for large scale task-shifting efforts to address the dearth of psychiatric service providers were first noted in 2012 [47]. In the 9 years since, several systematic reviews have concluded that task-shifting alone is not sufficient to bridge the mental health disorder treatment gap in LMICs, noting that the implementation strategy requires context-specific adaptations [12, 48]. Reviews of implementation strategies are bolstered by research efforts into the barriers and facilitators related to their implementation across contexts. This study adds to the knowledge base regarding barriers and facilitators to task-shifted integrated depression services in LMIC contexts.

## Data Availability

The datasets used and/or analysed during the current study are available from the corresponding author and the study principal investigator who is the co-author of this manuscript. This data can be realized upon request.

## Abbreviations

DMO: District Medical Officer
IDIs: In-depth interviews
IRBs: Institutional Review Boards
LMICs: Low and middle-income countries
MoH: Ministry of Health
NHSRC: National Health Science Research Committee
NCD: Non-communicable diseases’
PHQ: Patient Health Questioner
